# Association of body composition fat parameters and breast density in mammography by menopausal status

**DOI:** 10.1038/s41598-022-26839-y

**Published:** 2022-12-23

**Authors:** Ajung Chu, Pamela Sung, Jongyoon Lee, Jong-Ho Cheun, Ki-Tae Hwang, Kooklae Lee, Jiwon Kim, Jibong Jeong

**Affiliations:** 1 Department of Radiology, Seoul Metropolitan Government Seoul National University Boramae Medical Center, Seoul, Republic of Korea; 2 Department of Surgery, Seoul Metropolitan Government Seoul National University Boramae Medical Center, Seoul, Republic of Korea; 3 Division of Gastroenterology, Department of Internal Medicine, Seoul Metropolitan Government Seoul National University Boramae Medical Center, 20, Boramae‐ro 5‐gil, Dongjak‐gu, Seoul, 07061 Republic of Korea; 4 Department of Surgery, Seoul National University College of Medicine, Seoul, Republic of Korea

**Keywords:** Biomarkers, Risk factors

## Abstract

We investigated the relationship between body fat-driven obesity and breast fat density in mammography according to menopausal status. We retrospectively analyzed 8537 women (premenopausal, n = 4351; postmenopausal, n = 4186). Body fat parameters included BMI (body mass index), waist circumference (WC), waist-hip ratio (WHR), fat mass index (FMI), Percentage of body fat (PBF), and visceral fat area (VFA). Body fat-driven obesity was defined as follows: overall obesity, BMI ≥ 25 kg/m^2^; central obesity, WC > 85 cm; abdominal obesity, WHR > 0.85; excessive FMI, the highest quartile (Q4) of FMI; excessive PBF, the highest quartile (Q4) of VFA; visceral obesity, and the highest quartile (Q4) of VFA). Breast density was classified according to BI-RADS (grade a, b, c, and d), which defined as an ordinal scale (grade a = 1, grade b = 2, grade c = 3, and grade d = 4). All body fat-driven obesity parameters were negatively associated with the grade of breast density in both groups of women (*p* < 0.001): The more fatty parameters are, the less dense breast is. In multivariable binary logistic regression, all body fat-driven obesity parameters also showed a negative association with grade d density (vs. grade a, b, or c). In premenopausal women, BMI was a more associated parameter with grade d density than those of the other fat-driven parameters (OR 0.265, CI 0.204–0.344). In postmenopausal women, WC was more associated with grade d density than the others (OR 0.315, CI 0.239–0.416). We found that BMI, WC, WHR, FMI, PBF and VFA were negatively correlated with dense breast, and the association degree pattern between body fat-driven obesity and dense breast differs according to menopausal status.

## Introduction

Breast density in mammography reflects the proportion of fibroglandular tissue and fat in the breast. This is considered as one of the strongest markers for breast cancer risk in both premenopausal women and postmenopausal women^1,2^. Mammographic patterns and breast cancer risks are correlated in the general population, demonstrating strong linear trends for percentage density. Even in high-risk populations, previous studies have also shown that mammographic density is a predictor for breast cancer risk^3,4^.

Breast density is considered to correlate closely with body fat, but previous studies have shown inconsistent results. Data from a UK case control study and a Norwegian cohort study show that BMI is positively correlated with non-dense volumes, and density percentage and dense volume are positively associated with breast cancer risk^5^. A cohort study investigated the association of the interaction between mammographic breast density and BMI with breast cancer risk in Korean women and suggested that breast density and BMI interact synergistically to augment breast cancer risk, especially in postmenopausal women^4^. These correlations between BMI and breast cancer can result from the inflammatory effects of adiposity, which are mediated through increases in breast density. There is another theory that body fat aromatase activity can cause endogenous estrogens that promote breast tissue proliferation^6,7^. However, previous studies utilized BMI as an indicator of body composition, calculated as weight in kg/(height in m)^2^ because of its ease of measurement and calculation. However, weight may differ according to one’s body composition. BMI does not differentiate between body fat and lean mass^8^. Recent studies regarding obesity have shown differences between low body mass index and high body fat percentages among Asians^9^. These show that the relationship between body fat and BMI differs between Asians and Caucasians. The location of body fat also differs between people. Women of the same weight and height can have different breast densities. Considering this, it would be valuable to study the relationship between breast density and the body composition parameters.

In this study, we investigated the correlation of body composition parameters, including body mass index, waist circumference, waist-hip ratio, percentage of body fat, fat mass index, and visceral fat area, with breast densities, according to menopausal status.

## Materials and methods

### Study population

**Figure 1:**
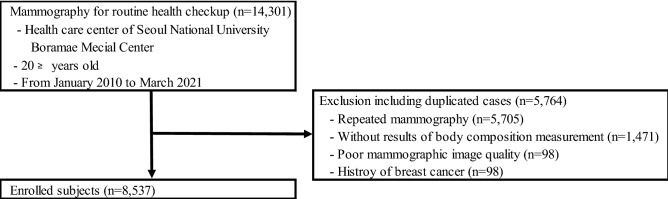
Enrollment flow chart of patients.

This single-center retrospective study was conducted at a health care center on women who had visited the hospital for routine checkups. We did 14,301 mammograms between January 2010 and March 2021 (Fig. 1). We excluded patients who had had repeated mammography (n = 5705), did not have body composition measurement results (n = 1471), had poor mammographic image quality (n = 98), or a history of breast cancer (n = 98). There were patients conforming to more than one exclusion criterion. In all, we excluded 5764 patients and enrolled 8537 patients for analysis. In patients with repeated mammograms, we used the initial mammography for analysis. We conducted our study in accordance with the Declaration of Helsinki, and the study protocol was approved by the Institutional Review Boards of the Seoul Metropolitan Government Seoul National University Boramae Medical Center (approval number: 20-2021-113). Obtaining informed consent was waived by the Institutional Review Boards of the Seoul Metropolitan Government and the Seoul National University Boramae Medical Center because of our study’s retrospective design and the routine nature of data collected.

### Clinical data

The participants had visited our health care center following an overnight 12-h fast. Clinical information and blood laboratory data were collected during the health checkup. Clinical information was collected for the following parameters: age, sex, systolic and diastolic blood pressure (BP), smoking, alcohol intake, and medical history, including hypertension (HT), diabetes mellitus (DM), and dyslipidemia (DL). HT was defined as systolic BP ≥ 140 mmHg, diastolic BP ≥ 90 mmHg, or the use of antihypertensive medications. DM was defined as fasting plasma glucose ≥ 126 mg/dL, glycated hemoglobin level ≥ 6.5%, or the use of antidiabetic medications including insulin. DL was defined as a TG (triglyceride) level ≥ 150 mg/dL, HDL-C < 50 mg/dL, or the use of medications. The following laboratory blood investigations had been done: total cholesterol, high-density lipoprotein cholesterol (HDL-C), low-density lipoprotein cholesterol (LDL-C), triglycerides (TG), glucose, aspartate aminotransferase (HbA1c), and C-reactive protein (CRP). Medical history including obstetric gynecology—menarche, menopause, delivery—and social history—tobacco use, alcohol consumption—were also collected using a questionnaire on routine checkup.

### Body fat parameters

Height and body weight were measured on the day of the health checkup. BMI was calculated as body weight divided by height squared (kg/m^2^). A trained nurse used a tape measure to measure the WC just above the standing patient’s hip bone as the patient exhaled. During the measurement, the tape measure was held flat against the body, not too tightly, in order to take a reading. Hip circumference was measured around the widest portion of the buttocks, with the tape measure parallel to the floor. WHR was calculated as WC/hip circumference. Body composition analysis was performed using the Inbody 720 (Biospace Co., Seoul, Korea) by a trained nurse following the manufacturer’s protocol^10^. Using the Inbody 720, body composition fat parameters [percent body fat (PBF, %), fat mass index (FMI, kg/m^2^), and visceral fat area (VFA, cm^2^)] were automatically calculated. The body composition parameter was divided into four parts using the quartile of each body fat parameter as follows. The PBF was divided into quartiles: quartile 1 (< 27.80%, Q1), quartile 2 (27.80–32.40%, Q2), quartile 3 (32.40–36.40%, Q3), and quartile 4 (≥ 36.40 mg/dL, Q4). The FMI level was divided into quartiles: quartile 1 (< 5.74 kg/m^2^, Q1), quartile 2 (5.74–7.21 kg/m^2^, Q2), quartile 3 (7.21–8.89 kg/m^2^, Q3), and quartile 4 (≥ 8.89 kg/m^2^, Q4). The VFA level was divided into quartiles: quartile 1 (< 58.10 cm^2^, Q1), quartile 2 (58.10–75.0 cm^2^, Q2), quartile 3 (75.0–96.30 cm^2^, Q3), and quartile 4 (≥ 96.30 cm^2^, Q4).

### Body fat-driven obesity parameters

We transformed numeric body fat parameters into binary body fat-driven obesity to clarify the obesity related to body fat parameters and the grade of dense breast as follows: overall obesity as BMI^11^ ≥ 25 kg/m^2^; central obesity as WC ≥ 85 cm^11^; abdominal obesity as WC > 0.85^12^; excess PBF as the highest quartile 4 (Q4) of PBF; excess FMI as the highest quartile 4 (Q4) of FMI; visceral obesity as the highest quartile 4 (Q4) of VFA.

### VFA

VFA measurements using the Inbody 720 have been reported to correlate well with the results of computed tomography (CT)^13^. In our study group, there was 580 subjects who underwent CT examination. Pearson’s bivariate correlation analysis showed a significant and strong correlation between VFA measured by Inbody 720 and by CT (r = 0.724, P < 0.001) (Supplementary Fig. S1).

### Breast density assessment

Breast density was interpreted visually by experienced radiologists with at least five years of experience. Radiologists of our center routinely graded dense breast on mammography into the four individual BI-RADS grades (grades a, b, c, and d)^14^. These are: (a) the breasts are almost entirely fatty; (b) there are scattered areas of fibroglandular density; (c) the breasts are heterogeneously dense, which may obscure small masses; (d) the breasts are extremely dense, which lowers the sensitivity of mammography. To explain baseline characteristics, grades a/b were described as fatty breast, and grades c/d were described as dense breast. Also, the breast density was used as a synonym for the BI-RADS grading system according to sentence flow.

### Statistical analysis

Continuous variables are expressed as mean ± standard deviation, and categorical variables are presented as numbers and percentages. Descriptive statistics were used to analyze the baseline characteristics of the study population. Comparison of characteristics and variables among groups with different grades of BI-RADS was performed via one-way analysis of variance (ANOVA). The association trend between body fat parameters and breast density was analyzed using linear-by-linear association, and nonparametric measures of correlation between body fat parameters and breast density were studied with Spearman’s rho. We performed multivariable binary logistic regression analyses to observe the role of body fat parameters as risk factors for grade d density (vs. a or b or c). The following clinical covariates were adjusted in this multivariable analysis: age, delivery, smoking, alcohol, TG, HDL-C, fasting glucose, and blood pressure. For postmenopausal women, the duration after menopause was additionally adjusted. Statistical analyses were conducted using IBM SPSS version 27 software (IBM Corp., Armonk, NY, USA).

## Results

Clinical characteristics according to the grade of breast density were analyzed in two separate groups [premenopause (n = 4351) and postmenopause (n = 4186)].

In premenopausal women, we evaluated clinical characteristics according to breast density. There were 462 patients with fatty breasts (a at 72 and b at 390, 10.6% of premenopausal women), 2291 patients with grade c (52.7%), and 1598 patients with grade d (36.7%). Patients with fatty breast were older (mean age: 42.0 ± 5.8) than those with grade c (mean age: 40.8 ± 5.8) or d (mean age: 38.6 ± 6.1) and associated with longer sex hormone exposure than patients with dense breasts. There were higher percentage of parous women (women who had experience with delivery, including normal or preterm delivery, regardless of live birth) in the grade a/b breast group (80.5%) than in the grade c/d breast group (c, 71.6%; d, 56.1%). Other medical conditions, including high blood pressure, diabetes, dyslipidemia, and metabolic syndrome levels were significantly higher in the fatty breast group. Body fat parameters were always highest in the fatty breast and lowest in extremely dense breast groups (grade d) (Table 1).

**Table 1:** Baseline characteristics according to grade of breast density in premenopausal and postmenopausal women.

Characteristics	Premenopausal women					Postmenopausal women				
	Total*	a/b*	c*	d*	*p†*	Total*	a/ b*	c*	d*	*p*^†^
	(n = 4351)	(n = 462)	(n = 2291)	(n = 1598)		(n = 4186)	(n = 2196)	(n = 1722)	(n = 268)	
Age (years, M ± SD)	40.1 ± 6.04	42.0 ± 5.84	40.78 ± 5.82	38.57 ± 6.06	< 0.001	60.55 ± 7.39	63.08 ± 7.8	57.97 ± 5.77	56.37 ± 5.38	< 0.001
**Smoking status**					0.047					< 0.001
Never, n (%)	3877 (89.1)	398 (86.1)	2050 (89.5)	1429 (89.4)		3955 (95.4)	2100 (95.6)	1644 (95.5)	251 (93.7)	
Past, n (%)	219 (5.0)	23 (5.0)	111 (4.8)	85 (5.3)		92 (2.2)	45 (2.0)	41 (2.4)	6 (2.2)	
Current, n (%)	255 (5.9)	41 (8.9)	130 (5.7)	84 (5.3)		99 (2.4)	51 (2.3)	37 (2.1)	11 (4.1)	
Alcohol intake, n (%)	1369 (31.5)	118 (25.5)	695 (30.3)	556 (34.8)	< 0.001	669 (16.0)	260 (11.8)	339 (19.7)	70 (26.1)	< 0.001
Diabetes mellitus, n (%)	101 (2.3)	31 (6.7)	53 (2.3)	17 (1.1)	< 0.001	579 (13.8)	370 (16.8)	192 (11.1)	17 (6.3)	< 0.001
Hypertension, n (%)	463 (10.6)	85 (18.4)	278 (12.1)	100 (6.3)	< 0.001	1622 (38.7)	1034 (47.1)	537 (31.2)	51 (19.0)	< 0.001
Dyslipidemia, n (%)	368 (8.5)	88 (19.0)	213 (9.3)	67 (4.2)	< 0.001	861 (20.6)	539 (24.5)	296 (17.2)	26 (9.7)	< 0.001
**Body fat parameter**										
BMI (kg/m^2^, M ± SD)	22.1 ± 3.32	25.08 + 4.75	22.52 ± 2.98	20.63 ± 2.39	< 0.001	23.57 ± 3.17	24.35 ± 3.22	22.92 ± 2.83	21.36 ± 2.84	< 0.001
WC (cm, M ± SD)	74.48 ± 8.92	80.85 + 11.71	75.81 ± 8.23	70.73 ± 7.21	< 0.001	79.95 ± 8.88	82.0 ± 8.95	78.4 ± 8.06	73.12 ± 7.9	< 0.001
WHR (ratio, M ± SD)	0.85 ± 0.05	0.88 + 0.05	0.85 ± 0.04	0.83 ± 0.04	< 0.001	0.88 ± 0.06	0.89 ± 0.06	0.86 ± 0.06	0.83 ± 0.05	< 0.001
FMI (kg/m^2^. M ± SD)	6.94 ± 2.37	8.94 ± 3.36	7.26 ± 2.11	5.89 ± 1.78	< 0.001	8.13 ± 2.46	8.73 ± 2.52	7.64 ± 2.18	6.33 ± 2.13	< 0.001
PBF, (%, M ± SD)	30.68 ± 6.072	34.67 ± 6.86	31.7 ± 5.47	28.06 ± 5.56	< 0.001	33.81 ± 6.25	35.18 ± 6.06	32.82 ± 5.86	28.94 ± 6.58	< 0.001
VFA (cm^2^, M ± SD)	69.93 ± 29.72	92.39 ± 41.71	72.94 ± 24.9	59.11 ± 27.18	< 0.001	91.01 ± 37.1	99.53 ± 38.11	83.82 ± 34.27	67.38 ± 23.96	< 0.001
**Obstetric history**										
Age at menarche (yr)	13.45 ± 1.89	13.62 ± 1.81	13.46 ± 1.87	13.4 ± 1.94	0.093	15.35 ± 2.24	15.68 ± 1.93	15.0 ± 2.36	14.92 ± 3.18	< 0.001
Parous^‡^, n (%)	2910 (66.9)	372 (80.5)	1641 (71.6)	897 (56.1)	< 0.001	3759 (89.8)	2003 (91.2)	1534 (89.1)	222 (82.8)	< 0.001
Age at menopause (yr)	NA	NA	NA	NA	NA	49.96 ± 4.72	49.85 ± 4.89	50.06 ± 4.53	50.23 ± 4.5	0.243
Postmenopausal duration (yr)	NA	NA	NA	NA	NA	10.59 ± 8.68	13.23 ± 9.14	7.91 ± 7.09	6.14 ± 6.78	< 0.001
HRT (n = 3466)	NA	NA	NA	NA	NA					< 0.001
Never, n (%)	NA	NA	NA	NA		2962 (85.5)	1593 (88.0)	1189 (82.6)	180 (83.3)	
Past, n (%)	NA	NA	NA	NA		300 (8.7)	131 (7.2)	150 (10.4)	19 (8.8)	
Current, n (%)	NA	NA	NA	NA		204 (5.9)	87 (4.8)	100 (6.9)	17 (7.9)	

Values are presented as mean (M) + standard deviation (SD) for continuous variables or n (%) for categorical variables.

*a, b, c, and d mean grades of mammographic breast density according to BI-RADS in premenopausal and postmenopausal women and Total is number of total cases in both groups.

^†^p values by ANOVA test for continuous variables and Chi square test for categorical variables.

^‡^Parous, parous women who had experience with delivery, including normal or preterm delivery, regardless of live birth.

*BMI* body mass index, *WC* waist circumference, *WHR* waist-hip ratio, *FMI* fat mass index, *PBF* percent body fat, *VFA* visceral fat area, *HRT* hormone replacement therapy, *NA* not applicable.

Over half of the postmenopausal women had fatty breasts (a at 832 and b at 1364, 52.5% of postmenopausal women) and the fatty breast group’s mean age was highest (mean age: 63.08 ± 7.8) in all density groups. Also, fatty breast population had longer postmenopausal duration than dense breast (13.23 ± 9.14 vs. c, 7.91 ± 7.09; d, 6.14 ± 6.78). The fatty breast group had higher percentage of parous women (91.2%), and the extremely dense group had lower percentage of parous women (82.8%), as did the premenopausal women. Hormone replacement therapy was more common in the dense breast groups (grade c and grade d, 17.3% and 16.7%) than in the fatty breast groups (12.0%). The fatty breast groups had higher blood pressures, diabetes, dyslipidemia, and metabolic syndrome. Their body fat parameters also were the highest of the three groups (Table 1).

To analyze the association trend of body fat-driven obesity parameters according to the grade of dense breast, we performed a linear-by-linear analysis (Table 2). All body fat-driven obesity parameters had significantly negative association trends according to the increase in their grades in both premenopausal and postmenopausal women (Table 2).

**Table 2:** Association trend of body fat-driven obesity parameters according to grade of breast density in premenopausal and postmenopausal women.

Parameters	Premenopausal women						Postmenopausal women			
	Total*	a/b*	c*	d*	*p* for trend^†^	Total*	a/b*	c*	d*	*p* for trend^†^
	(n = 4351)	(n = 462)	(n = 2291)	(n = 1598)		(n = 4186)	(n = 2196)	(n = 1722)	(n = 268)	
Overall obesity, n (%)	687 (15.8)	202 (43.7)	407 (17.8)	78 (4.9)	< 0.001	1242 (29.7)	864 (39.3)	349 (20.3)	29 (10.8)	< 0.001
Central obesity, n (%)	532 (12.2)	148 (32.0)	316 (13.8)	68 (4.3)	< 0.001	1235 (29.5)	811 (36.9)	398 (23.1)	26 (9.7)	< 0.001
Abdominal obesity, n (%)	2027 (46.6)	356 (77.1)	1258 (54.9)	413 (25.8)	< 0.001	3052 (72.9)	1810 (82.4)	1133 (65.8)	109 (40.7)	< 0.001
Excess PBF, n (%)	717 (16.5)	185 (40.0)	427 (18.6)	105 (6.6)	< 0.001	1450 (34.6)	948 (43.2)	464 (26.9)	38 (14.2)	< 0.001
Excess FMI, n (%)	717 (16.5)	185 (40.0)	427 (18.6)	105 (6.6)	< 0.001	1450 (34.6)	948 (43.2)	464 (26.9)	38 (14.2)	< 0.001
Visceral obesity, n (%)	586 (13.5)	169 (36.6)	338 (14.8)	79 (4.9)	< 0.001	1553 (37.1)	1056 (48.1)	465 (27.0)	32 (11.9)	< 0.001

*a, b, c, and d mean grade of mammographic breast density according to BI-RADS in premenopausal and postmenopausal women.

and Total is number of total cases in both groups.

^†^p values by linear-by-linear association.

Overall obesity, BMI ≥ 25 kg/m^2^; Central obesity, waist circumference (WC) > 85 cm; Abdominal obesity, waist-hip ratio (WHR) > 0.85; Excessive PBF, PBF ≥ 36.4% which is the highest quartile (Q4) of percent body fat (PBF); Excessive FMI, FMI ≥ 8.85 kg/m^2^ which is the highest quartile (Q4) of fat mass index (FMI); Visceral obesity, VFA ≥ 96.3 cm^2^ which is the highest quartile (Q4) of visceral fat area (VFA).

To see the correlation between the body fat parameters and the ordinal grade of dense breast, we analyzed the fat parameters and the grade of dense breast with a nonparametric correlation measure. WHR demonstrated the most favorable correlation with the fat parameter among the parameters, which was moderately correlated with the grade of dense breast in premenopausal women (Spearman's rho = − 0.413) and weakly correlated with the grade of that in postmenopausal women (Spearman's rho = − 0.318). When the body fat parameters, including BMI, WC, FMI, PBF, and VFA were relatively higher, the grade of dense breast was relatively lower in both the premenopausal and postmenopausal group. Each fat parameter's absolute correlation magnitude was always higher in premenopausal women than that in postmenopausal women (Table 3).

**Table 3:** Correlations between body fat parameters and grade of breast density.

Parameters	Premenopausal women		Postmenopausal women	
	(n = 4351)		(n = 4186)	
	ρ*	*p*^†^	ρ*	*p*^†^
BMI (kg/m^2^)	− 0.408	< 0.001	− 0.284	< 0.001
WC (cm)	− 0.352	< 0.001	− 0.263	< 0.001
WHR (ratio)	− 0.413	< 0.001	− 0.318	< 0.001
PBF (%)	− 0.351	< 0.001	− 0.25	< 0.001
FMI (kg/m^2^)	− 0.395	< 0.001	− 0.277	< 0.001
VFA (cm^2^)	− 0.379	< 0.001	− 0.311	< 0.001

*Spearman’s rank correlation coefficient.

^†^p values by nonparametric measure of correlation.

*BMI* body mass index, *WC* waist circumference, *WHR* waist-hip ratio, *PBF* percent body fat, *FMI* fat mass index, *PBF* percent body fat, *VFA* visceral fat area.

The grades of breast density were ordinal (grade a = 1, grad b = 2, grade c = 3, and grade d = 4).

After controlling for potential confounders, the adjusted OR to grade d (vs. grade a, b, or c) density was significantly smaller than 1 in all body fat-driven obesity parameters, including overall obesity, central obesity, abdominal obesity, excess PBF, excessive FMI, and visceral obesity. In postmenopausal women, we also included duration following menopause as a confounding factor. In premenopausal women, overall obesity was more associated with grade d density (adjusted OR, 0.265; 95% CI, 0.204–0.344), whereas abdominal obesity was more associated with grade d density than the others (adjusted OR, 0.315; 95% CI, 0.239–0.416) (Table 4).

**Table 4:** Risk of the grade d (vs. grade a, b, or c) density according to body fat-driven obesity parameters using multivariable binary logistic regression.

Parameters	Premenopausal women		Postmenopausal women	
	Adjusted OR (95% CI)	*p*	Adjusted OR (95% CI)	*p*
Overall obesity	0.265 (0.204–0.344)	< 0.001	0.456 (0.303–0.713)	< 0.001
Central obesity	0.316 (0.239–0.416)	< 0.001	0.348 (0.229–0.530)	< 0.001
Abdominal obesity	0.330 (0.285–0.383)	< 0.001	0.315 (0.239–0.416)	< 0.001
Excessive PBF	0.318 (0.252–0.400)	< 0.001	0.389 (0.271–0.558)	< 0.001
Excessive FMI	0.314 (0.247–0.399)	< 0.001	0.402 (0.277–0.583)	< 0.001
Visceral obesity	0.384 (0.296–0.500)	< 0.001	0.329 (0.223–0.485)	< 0.001

*OR* odds ratio, *CI* confidence interval.

Overall obesity, BMI ≥ 25 kg/m^2^; Central obesity, waist circumference (WC) > 85 cm; Abdominal obesity, waist-hip ratio (WHR) > 0.85; The grade d of breast density, is extremely dense breast of BI-RADS; Excessive PBF, PBF ≥ 36.4% which is the highest quartile (Q4) of percent body fat (PBF) with reference to Q1, Q2, or Q3; Excessive FMI, FMI ≥ 8.85 kg/m^2^ which is the highest quartile (Q4) of fat mass index (FMI) with reference to Q1, Q2, or Q3; Visceral obesity, VFA ≥ 96.3 cm^2^ which is the highest quartile (Q4) of visceral fat area (VFA) with reference to Q1, Q2, or Q3.

Multivariable logistic regression model was applied with grades a, b, and c of BI-RADS as reference and adjusted for age, parity, smoking, alcohol, TG, HDL-C, fasting glucose, systolic BP, and diastolic BP in premenopausal women.

## Discussion

Our results imply distinct impacts on various body fat parameters between premenopausal and postmenopausal women.

Mammographic density is a strong, independent risk factor for breast cancer^15–17^. Age-related breast density declines may seem to be paradoxical, as breast cancer incidence increases with age, but Pike and colleagues proposed a model to explain these results^16^. According to this model, the breast tissue aging diminishes with age, but the effect of age is cumulative over time, and, therefore, increases breast cancer risk. To understand the role of breast density, we have to understand the correlation between it and fat density. Previous studies about breast density and body fat used BMI as a body fat marker. Several studies have investigated the factors related to breast density and body fat, but the study samples were small and the results varied between studies^18–20^. A prospective cohort study of the Breast Cancer Surveillance Consortium (BCSC) reported heterogeneous or extreme breast density was associated with an increased risk of breast cancer in women 65–74 years of age and women aged 75 years or older with a BMI of 25 or greater. They found breast density is associated with breast cancer regardless of the BMI, but they were not looking at the association between BMI and breast density. Further, they found that extreme dense breasts were more likely to be normal weight, but this was an unadjusted model and, furthermore, in this group there was a higher proportion of Asian women^21^. These inconsistent results suggest the need to evaluate precise parameters regarding mammographic density. One study looked at the skeletal muscle mass index to reflect a relatively accurate fat-to-muscle ratio^22^. In a cross-sectional study of 143,456 women, the odds ratio for breast density was between the highest and lowest SMI (skeletal muscle index) quartiles at 2.65 for premenopausal women and at 2.39 for postmenopausal women. One of the strongest factors affecting breast density is menopausal status. In a study of menopausal transition, BMI and density, the prevalence of dense breasts was negatively associated with increasing menopausal stages. In overweight women (BMI > 23 kg/m^2^), it was more profound^23^. Our study showed all body fat parameters are negatively correlated to breast density, regardless of menopausal status. But in premenopausal women, BMI was more correlated with breast density, compared with the other fat parameters, whereas WHR was more correlated with breast density compared with those in postmenopausal women. So, in premenopausal women we need to focus more on BMI than other parameters whereas, in postmenopausal women, the focus should be on WHR. Those differences may result from the fat mass changes in postmenopausal women. A meta-analysis showed a central fat increment following menopause^24^. This study showed a significant decrease in total leg fat and increase in central and total body fat following menopause. Our results also suggest that in menopausal women, breast fat distribution is more affected by central fat (correlated with WHR) than total body fat or BMI. There are several studies that have examined the association between body fat parameters and breast density. We summarized the results of these studies (Supplementary Table S1). The results are inconsistent and do not conclusively establish an association between breast density with body fat parameters. In addition, body fat parameters used in most of these studies were anthropometric indices, mainly BMI. Few studies used waist to hip ratio or skeletal muscle mass index. On the other hand, in our study, we used multiple parameters including PBF, FMI, and VFA, and the number of study subjects was very large with 8537 subjects.

Obesity is a well-known risk factor for the growth and progression of breast cancer. But the mechanism by which adiposity affects breast cancer is still unknown. Many studies have revealed new insights into this link. The Premenopausal Breast Cancer Collaborative Group reported an inverse association of BMI and age and subsequent breast cancer risk in premenopausal women^25^. Their results showed an increased adiposity by BMI, associated with a reduced risk of premenopausal breast cancer. They also observed that the strongest risk association (a 4.2-fold risk gradient between underweight versus obese women) was for BMI in early adulthood (ages 18–24 years). Another group also reported decreased risk of breast cancer in the overweight and obesity groups^26^. The authors suggested that these results were partly attributed to a higher rate of amenorrhea in obese women, consequently decreasing circulating estrogen levels. The International Agency for Research on Cancer (IARC) has reported that obesity (as determined by BMI) is associated with breast cancer (relative risk 1.1 for the highest BMI category evaluated compared to normal BMI) only in postmenopausal women, especially for estrogen receptor-positive tumors^27^. Waist circumference and body weight gains in adulthood were positively associated with the risk of postmenopausal breast cancer. But in premenopausal women, BMI and breast cancer were negatively correlated. However, data on associations of breast cancer with waist circumference and body weight gain were inconsistent. In a systematic review of BMI and cancer incidence, Asia-Pacific populations showed a strong association in premenopausal (*p* = 0.009) and postmenopausal (*p* = 0.06) cancers; whereas, in the general population (North American, European and Australian, and Asia-Pacific), postmenopausal breast cancer showed only weak positive associations with BMI^28^. Obesity and dense breasts are the two most well-known risk factors for breast cancer. But breast density and BMI are inversely correlated and act as confounders to each other’s effect^2,17^. Our results suggest that we need to focus on the other effects that BMI and central fat have on breast density in menopausal women. To reduce breast cancer risk, both breast density and BMI need to be modified, tailored to the individual’s hormonal status.

Our study has several limitations. First, as this is a single center cross-sectional study, our results only show correlations between body fat parameters and breast density. The underlying physiological pathway is not fully understood. Second, we used BIA to measure body fat parameters. Although all body fat parameters correlated equally with breast density, these have not been widely used to assess body composition. Further research is needed to determine how breast density pathophysiology correlates with BMI and menopause. Third, we used mammographic density in BI-RADS category. Recent studies suggest the absolute volume of fibroglandular tissue in breasts may play an important role in breast density and obesity. Further studies using MRI or ultrasound may allow for the use of volume data to reveal a correlation.

## Conclusions

In summary, our results show that all body fat parameters are negatively correlated with the grade of breast density, regardless of menopausal status. Overall obesity (as represented by BMI) is a more associated risk factor than the other parameters in premenopausal women. But, central obesity (as represented by WHR) is a more associated risk factor than the others in postmenopausal women. The pattern of degree of association among body fat parameters and breast density differs according to menopausal status.

## Supplementary Information

Supplementary Information.

## Data Availability

All data underlying the results are available as part of the article. Raw data that support the findings of this study are available from the corresponding author, upon reasonable request.

## Abbreviations

BMI: Body mass index
CI: Confidence interval
FMI: Fat mass index
IRB: Institutional Review Board
OR: Odds ratio
PBF: Percent body fat
VFA: Visceral fat area
WC: Waist circumference
WHR: Waist–hip ratio
