# Supplementary material for: Association of body composition fat parameters and breast density in mammography by menopausal status
Source: Sci Rep. 2022 Dec 23;12:22224. doi: 10.1038/s41598-022-26839-y (PMC9789058; doi:10.1038/s41598-022-26839-y)
Supplement: Supplementary file 1 — Supplementary Information. [file 41598_2022_26839_MOESM1_ESM.docx]

**Supplementary data**

**Supplementary Figure S1. Correlation between VFA measured by InBody 720 and by computed tomography**

**
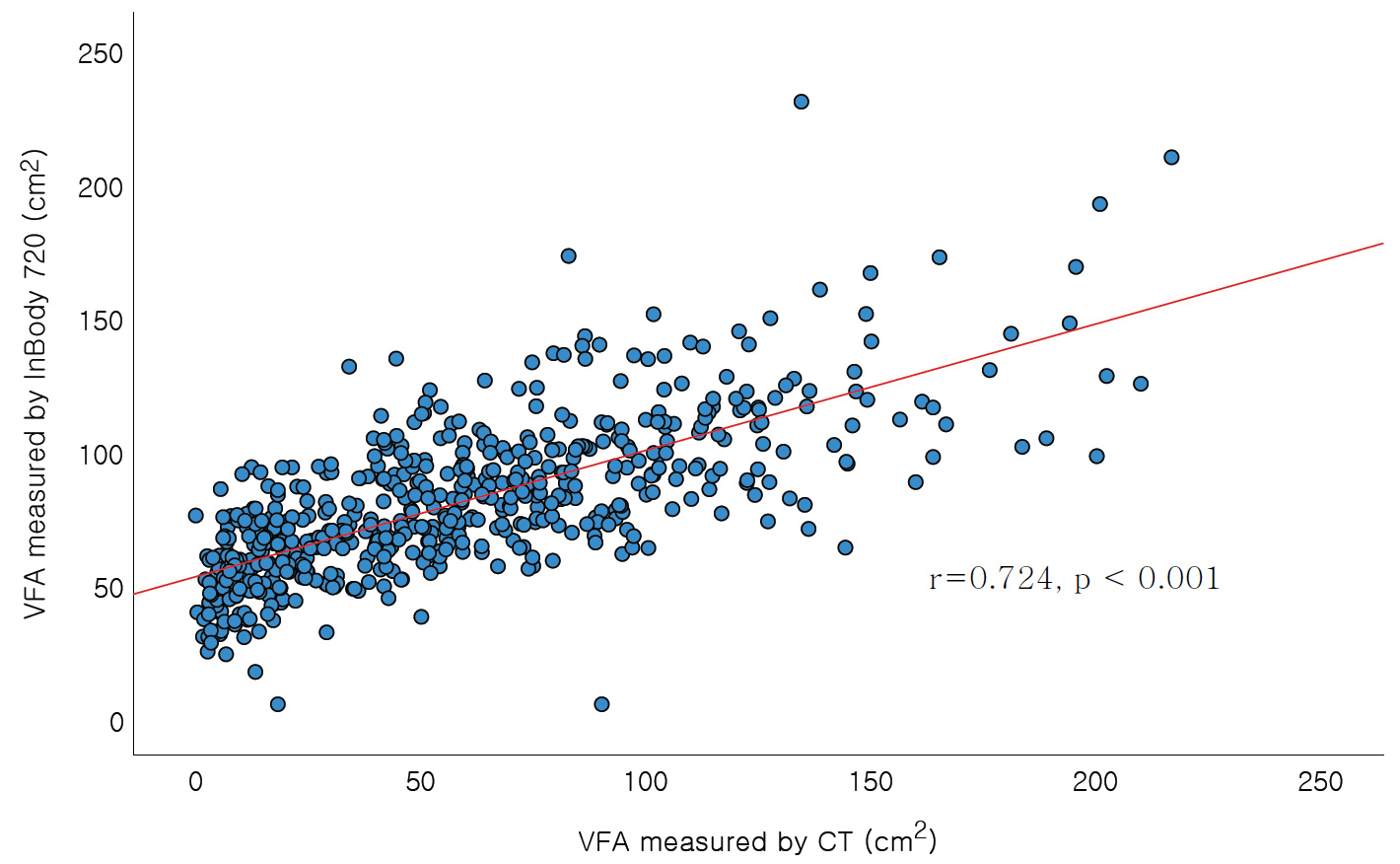
**

**Supplementary Table S1. Summary of studies showing the association between parameters of breast density**

| **Source (year)** | **Journal type** | **Number of subjects** | **Measure of body fat** | **Measure of density** | **Main result** | **Summary of findings** |
| --- | --- | --- | --- | --- | --- | --- |
| Soguel et al. (2021)^1^ | Review article | 31 studies | Variable | BI-RADS category, areas or volumes | Negative association | It is not surprising that static measures of adiposity were associated positively with absolute non-dense area. |
| Hjerkind et al. (2018)^2^ | Original article | 46,428 subjects | BMI | VMD | Negative association with percent VMD, Positive association with absolute VMD | BMI was positively associated with absolute VMD, with 1.5 times higher VMD in women in the highest, relative to those in the lowest, BMI category [58.4 cm3 (95% CI, 57.4–59.5 cm3] vs. 37.9 cm3 (95% CI, 37.1–38.8 cm3)]. |
| Lee et al. (2021)^3^ | Original article | 143,456 subjects | SMI, BMI | BI-RADS category | Negative association with BMI and WC, Positive association with SMI | After adjusting for confounding factors including BMI, the odds ratios for MD for the dense breasts were between the highest and lowest quartiles of SMI at 2.65 for premenopausal women and 2.39 for postmenopausal women. |
| Boyd et al. (1998)^4^ | Original article | 273 premenopausal women | Weight, skinfold thickness | Dense and non-dense measurement |  | All skinfold  measures. and their sum were strongly and positively correlated  with the total area and the area of non-dense tissue, and negatively correlated with the  percent area of dense tissue. |
| Haars et al.(2005)^5^ | Original article | 418 postmenopausal women | BMI | Amount of dense tissue and non-dense tissue | The percent dense tissue is negatively associated with BMI | In this study, the effect of BMI on the relative amount of density was entirely due to the relationship between BMI and non-dense tissue. |
| Irwin et al. (2013)^6^ | Original article | 522 postmenopausal women | BMI | Mammographic density | Negative association | We observed a statistically significant decline in percent density (p for trend = .0001), and mammographic dense area (p for trend = 0.0052), with increasing levels of BMI adjusted for potential covariates. |
| Soguel et al. (2016)^7^ | Original article | 1,435 subjects | BMI | Percent density, absolute dense area, and non-dense area | BMI and percent density or absolute dense area were inversely correlated, but BMI was positively correlated with the absolute dense area when adjusting for the absolute non-dense area. | Adiposity appears to be positively associated with both dense and non-dense areas following adjustment for each other. Our findings suggest a higher breast dense area among women who gained weight and that a minimum of breast fat may be needed to promote the proliferation of this fibroglandular tissue. |
| Pollan et al. (2012)^8^ | Original article | 3,584 subjects | BMI, abdominal fat distribution, adult weight gain | Mammographic density | WHR was inversely associated with MD, adult weight gain was positively associated with MD | Waist-to-hip ratio was inversely associated with MD, and the effect was more pronounced in pre-menopausal (OR = 0.53 per 0.1 units; 95 % CI = 0.42–0.66) than in post-menopausal women (OR = 0.73; 95 % CI = 0.65–0.82) (p of heterogeneity = 0.010). In contrast, adult weight gain displayed a positive association with MD, which was similar in both groups (OR = 1.17 per 6 kg; 95 % CI = 1.11–1.23). |

BMI, body mass index; WC, waist circumference; VMD, volumetric mammography density; SMI, skeletal muscle mass index; WHR, waist to hip ratio;

**References**

1. Soguel L﻿, Durocher F﻿, Tchernof A﻿, Diorio C﻿. Adiposity, breast density, and breast cancer risk: epidemiological and biological considerations. ﻿Eur J Cancer Prev. 2017;26(6):511-520. doi:10.1097/CEJ.0000000000000310

2. Hjerkind KV﻿, Ellingjord-Dale M﻿, Johansson ALV﻿, et al. Volumetric mammographic density, age-related decline, and breast cancer risk factors in a national breast cancer screening program. Cancer Epidemiol Biomarkers Prev. 2018;27(9):1065-1074. doi:10.1158/1055-9965.EPI-18-0151

3. Lee KH, Chae SW, Yun JS, Park YL, Park CH. Association between skeletal muscle mass and mammographic breast density. Sci Rep. 2021 Aug 18;11(1):16785. doi: 10.1038/s41598-021-96390-9. PMID: 34408263; PMCID: PMC8373895.

4. Boyd, N., Lockwood, G., Byng, J. et al. The relationship of anthropometric measures to radiological features of the breast in premenopausal women. Br J Cancer 78, 1233–1238 (1998). https://doi.org/10.1038/bjc.1998.660

5. Haars G, van Noord PA, van Gils CH, Grobbee DE, Peeters PH. (2005). Measurements of breast density: no ratio for a ratio. Cancer Epidemiol Biomarkers Prev 14 (Pt 1):2634–2640.

6. In this study, the effect of BMI on the relative amount of density is entirely due to the relation between BMI and nondense tissue.

7. Soguel L, Diorio C. (2016). Anthropometric factors, adult weight gain, and mammographic features. Cancer Causes Control 27:333–340.

8. Pollán M, Lope V, Miranda-García J, García M, Casanova F, Sánchez-Contador C, et al., DDM-Spain (2012). Adult weight gain, fat distribution and mammographic density in Spanish pre- and post-menopausal women (DDM-Spain). Breast Cancer Res Treat 134:823–838.
